# Supplementary material for: Genome-wide association study suggests that variation at the RCOR1 locus is associated with tinnitus in UK Biobank
Source: Sci Rep. 2021 Mar 19;11:6470. doi: 10.1038/s41598-021-85871-6 (PMC7979698; doi:10.1038/s41598-021-85871-6)

Genome-wide association study suggests that variation at the RCOR1 locus is associated with tinnitus in UK Biobank.

Helena R.R. Wells, Fatin N. Zainul Abidin, Maxim B. Freidin, Frances M.K. Williams and Sally J. Dawson.

Supplementary Figure 1. Locus plots for each of the 11 suggestive loci.

Supplementary Figure 2. Manhattan plot for genetic association analysis with severest tinnitus subgroup only

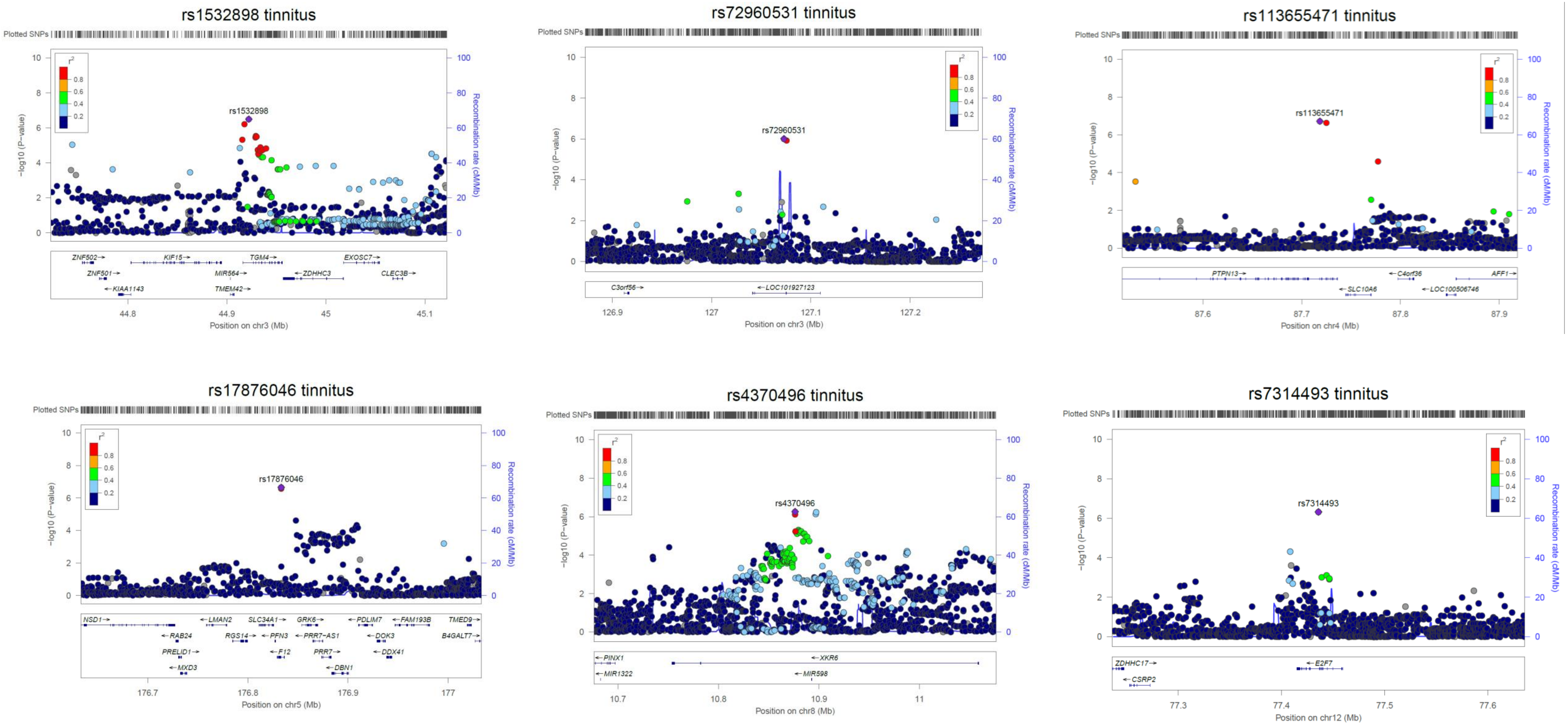
rs1532898 tinnitus
Plotted SNPs
-log10 (P-value)
Recombination rate (cM/Mb)
r2
rs1532898
ZNF502
ZNF501
KIAA1143
KIF15
MIR564
TMEM42
TGM4
ZDHHC3
EXOSC7
CLEC3B
44.8
44.9
45
45.1
Position on chr3 (Mb)
rs72960531 tinnitus
rs72960531
C3orf56
LOC101927123
126.9
127
127.1
127.2
Position on chr3 (Mb)
rs113655471 tinnitus
rs113655471
PTPN13
C4orf36
AFF1
SLC10A6
LOC100506746
87.6
87.7
87.8
87.9
Position on chr4 (Mb)
rs17876046 tinnitus
rs17876046
NSD1
RAB24
PRELID1
MXD3
LMAN2
RGS14
SLC34A1
PFN3
F12
GRK6
PRR7-AS1
PRR7
DBN1
PDLIM7
DOK3
DDX41
FAM193B
TMED9
B4GALT7
176.7
176.8
176.9
177
Position on chr5 (Mb)
rs4370496 tinnitus
rs4370496
PINX1
MIR1322
XKR6
MIR598
10.7
10.8
10.9
11
Position on chr8 (Mb)
rs7314493 tinnitus
rs7314493
ZDHHC17
CSRP2
E2F7
77.3
77.4
77.5
77.6
Position on chr12 (Mb)

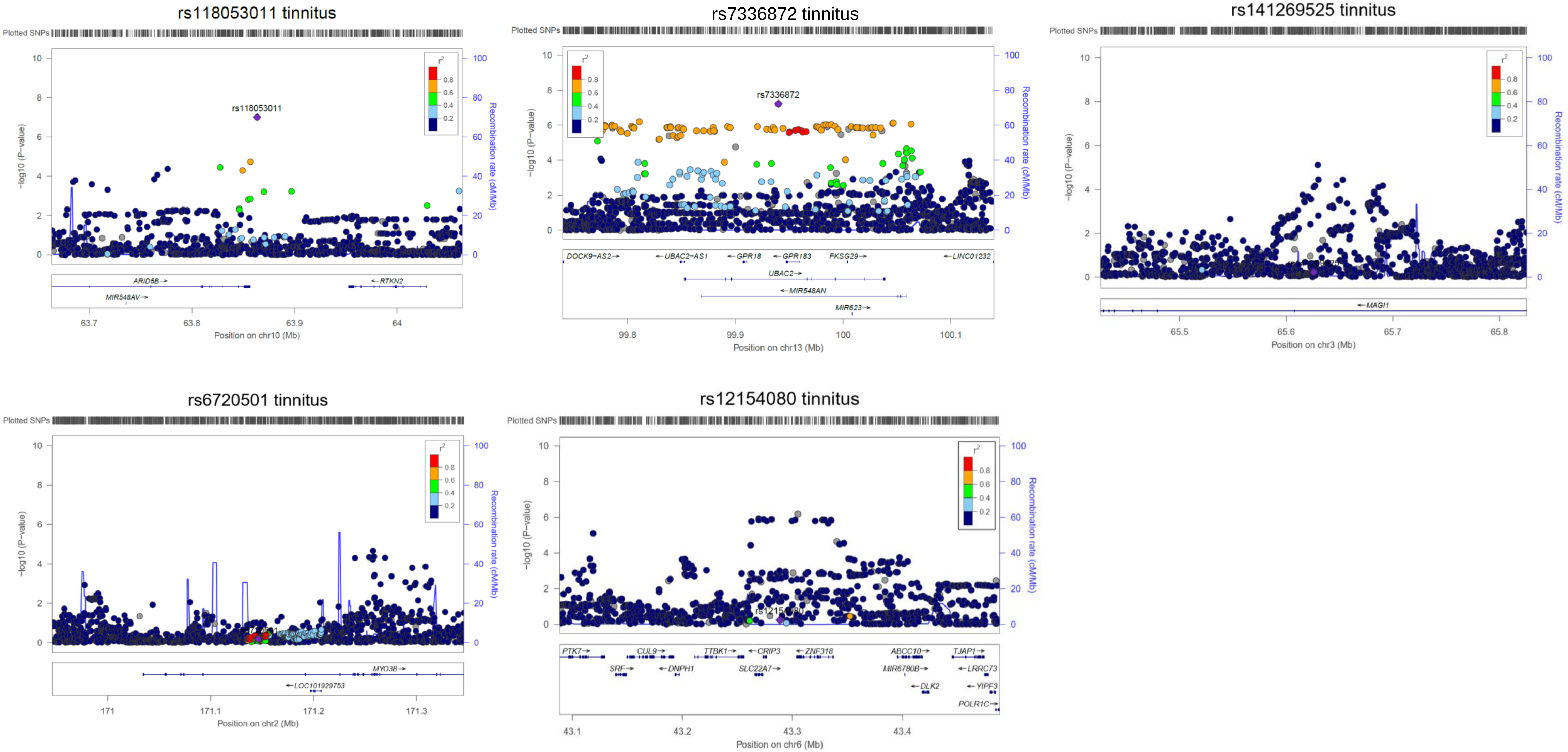

Supplementary Figure 1. Locus Plots for 11 loci at suggestive level associations with tinnitus in UKBB. Purple indicates lead independent SNP generated from GCTA-COJO conditional analysis. The colouring of remaining SNPs is based on the linkage disequilibrium (r^2) with the leads. Where LD information is not available, SNPs are coloured grey. The genes within the region are annotated and the direction of transcription is indicated by arrows. Plots were generated using LD Zoom and for 3 loci the lead SNPs were not available and for these the region around the closest available SNP in LD Zoom was plotted [rs6720501 for 2:171146084_CTT_C; rs1215408 for rs553448379; rs141269525 for rs557511691].

Supplementary Figure 2. Manhattan plot for genetic association analysis with severest tinnitus subgroup only
The red dotted line marks the genome-wide significance threshold of P<5E-08.

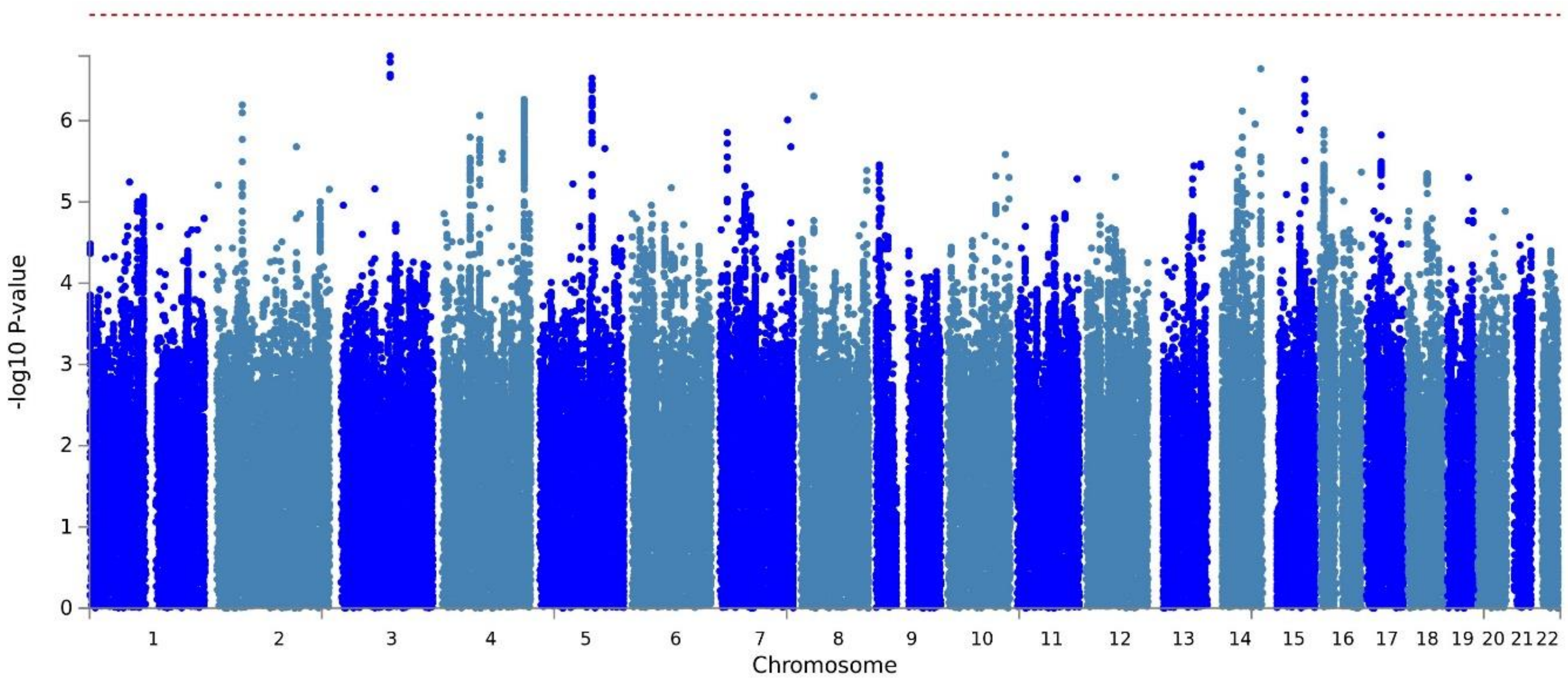

Supplement: Supplementary file 1 — Supplementary Information 1. [file 41598_2021_85871_MOESM1_ESM.pdf]
